# IL-1β promotes hypoxic vascular endothelial cell proliferation through the miR-24-3p/NKAP/NF-κB axis

**DOI:** 10.1042/BSR20212062

**Published:** 2022-01-18

**Authors:** Jiangnan Huang, Yumei Li, Zhiyuan Jiang, Lingjun Wu, Yueying Liu, Siwen Ma, Lang Li, Hui Wang

**Affiliations:** 1 Department of Hypertension, The First Affiliated Hospital of Guangxi Medical University, Nanning, China; 2 Department of Molecular Microbiology and Immunology, University of Southern California, Los Angeles, CA, U.S.A.; 3 School of Pharmacy, Guangxi Medical University, Nanning, China; 4 Department of Cardiology, The First Affiliated Hospital of Guangxi Medical University, Nanning, China

**Keywords:** hypoxic HUVECs, IL-1β, miR-24-3p, NF-kB, NKAP

## Abstract

**Purpose:** Our previous data indicated that miR-24-3p is involved in the regulation of vascular endothelial cell (EC) proliferation and migration/invasion. However, whether IL-1β affects hypoxic HUVECs by miR-24-3p is still unclear. Therefore, the present study aimed to investigate the role and underlying mechanism of interleukin 1β (IL-1β) in hypoxic HUVECs.

**Methods:** We assessed the mRNA expression levels of miR-24-3p, hypoxia-inducible factor-1α (HIF1A) and NF-κB-activating protein (NKAP) by quantitative real-time polymerase chain reaction (RT-qPCR). ELISA measured the expression level of IL-1β. Cell counting kit-8 (CCK-8) assays evaluated the effect of miR-24-3p or si-NKAP+miR-24 on cell proliferation (with or without IL-1β). Transwell migration and invasion assays were used to examine the effects of miR-24-3p or si-NKAP+miR-24-3p on cell migration and invasion (with or without IL-1β). Luciferase reporter assays were used to identify the target of miR-24-3p.

**Results:** We demonstrated that in acute myocardial infarction (AMI) patient blood samples, the expression of miR-24-3p is down-regulated, the expression of IL-1β or NKAP is up-regulated, and IL-1β or NKAP is negatively correlated with miR-24-3p. Furthermore, IL-1β promotes hypoxic HUVECs proliferation by down-regulating miR-24-3p. In addition, IL-1β also significantly promotes the migration and invasion of hypoxic HUVECs; overexpression of miR-24-3p can partially rescue hypoxic HUVECs migration and invasion. Furthermore, we discovered that NKAP is a novel target of miR-24-3p in hypoxic HUVECs. Moreover, both the overexpression of miR-24-3p and the suppression of NKAP can inhibit the NF-κB/pro-IL-1β signaling pathway. However, IL-1β mediates suppression of miR-24-3p activity, leading to activation of the NKAP/NF-κB pathway. In conclusion, our results reveal a new function of IL-1β in suppressing miR-24-3p up-regulation of the NKAP/NF-κB pathway.

## Introduction

Acute myocardial infarction (AMI) and heart failure (HF) are often the leading causes of death and disability worldwide, especially in developed countries [1–3]. AMI is an event of myocardial necrosis that may lead to functional loss in the diastolic and systolic regions and cause arrhythmias in patients [4]. In addition, AMI and ischemia–reperfusion injury dysfunction in the myocardium induce an acute inflammatory response, which activates the inflammasome and maturation of proinflammatory cytokines (e.g., interleukin 1β (IL-1β)) [5]. Therefore, investigating the molecular mechanisms responsible for hypoxic HUVECs progression remains crucial.

IL-1β is a critical proinflammatory cytokine involved in up-regulating adhesion molecules and metalloproteinases [6]. Thus, IL-1β plays an essential role in the development of atherosclerosis and other cardiovascular diseases. In addition, IL-1β induces a further loss of viable myocardium, promoting cardiac dilation and dysfunction in the subacute phase of AMI and suppressing cardiac contractility and β-adrenergic receptor responsiveness [7]. Therefore, inhibitors of the inflammasome or IL-1β administered during AMI have the potential to prevent adverse cardiac remodeling and HF.

MicroRNAs (miRNAs) are small noncoding RNA molecules approximately 22 nucleotides in length that can play essential roles in animals and plants [8]. As a class of negative regulatory molecules, miRNAs degrade or inhibit the translation of target mRNAs by binding to the 3′-untranslated region (3′-UTR) of the target mRNA [9]. It has been shown that miRNAs can regulate approximately 60% of human protein-coding genes [10]. Previous studies have shown that miRNAs are widely involved in cell proliferation, differentiation, and apoptosis [11]. Additionally, miRNAs are associated with human disease occurrence [12,13]. An increasing number of studies have shown that hundreds of miRNAs are involved in the development and progression of cardiovascular diseases [14], especially in myocardial infarction [15]. Recently, miR-24-3p was shown to play a vital role in cardiovascular diseases [16], especially angiogenesis [17]. miR-24-3p is derived from the miR-23a–27a–24-2 gene clusters, which have also been widely studied in cancer, such as lung cancer [18], liver cancer [19], breast cancer [20], and prostate cancer [21]. Moreover, our previous data indicated that miR-24 inhibits the proliferation and migration of HUVECs [22]. However, the potential molecular mechanisms of IL-1β cross-talk with miR-24-3p have not yet been fully elucidated.

Here, we further studied the roles of IL-1β in hypoxic HUVECs. First, our results showed that IL-1β promotes hypoxic HUVECs proliferation. Additionally, IL-1β promotes hypoxic HUVECs migration and invasion by down-regulating miR-24-3p. Next, we confirmed that NF-κB-activating protein (NKAP) is a direct target of miR-24-3p. Moreover, the silencing of NKAP expression was shown to account for the downstream effects of miR-24-3p in hypoxic HUVECs. Furthermore, both overexpression of miR-24-3p and silencing of NKAP suppress the NF-κB signaling pathway. In conclusion, our results reveal the miR-24-3p/NKAP/NF-κB axis and explain how IL-1β promotes proliferation and migration/invasion in hypoxic HUVECs.

## Materials and methods

### Blood collection

Peripheral blood samples were collected from 12 healthy donors and 12 AMI patients in PAXGene tubes according to the manufacturer’s instructions. Collection and use of blood samples were approved by the human research ethics committee of the First Affiliated Hospital of Guangxi Medical University and all subjects provided informed consent. Total RNA was extracted from 2.5 ml of blood with the PAXGene Blood miRNA Kit (Qiagen, U.S.A.), designed for the simultaneous isolation of small and large RNAs. RNA concentration and quality were assessed through a NanoDrop spectrophotometer (Thermo Scientific, U.S.A.). Then, reverse transcription was performed by using a cDNA Synthesis Kit (Invitrogen, U.S.A.). The stem-loop RT primer for miR-24-3p was GTCGTATCCAGTGCAGGGTCCGAGGTATTCGCACTG GATACGACCTGTTCC. The miRNA expression level was evaluated using an miRNA qPCR detection kit (Agilent, U.S.A.) according to the manufacturer’s protocol. The primers for miR-24-3p were as follows: miR-24-3p-F: 5′-GTCGTATCCAGTGCAGG GTCC-3′; miR-24-3p-R: 5′-AATCGGCGTGGCTCAGTTCAG-3′.

### Isolation of human circulating endothelial cells

Peripheral blood samples were collected from 12 healthy donors and 12 AMI patients. Human peripheral blood mononuclear cells (PBMCs) were isolated from donors using the Ficoll gradient (Histopaque-1077, Sigma) as previously described [23]. Then, human circulating endothelial cells (CECs) were also freshly isolated from human PBMCs with the CD31 MicroBead Kit (Miltenyi, #130-091-935).

### ELISA

Peripheral blood samples were collected from 12 healthy donors and 12 AMI patients in 1.5 ml tubes. The tubes were kept at room temperature for 15 min and then centrifuged at 12000×***g*** for 5 min. Serum was collected, and the ELISA was performed using 100 µl serum per well.

### Cell lines and miRNAs

The human endothelial cell line (HUVEC) and human coronary artery endothelial cell line (HCAEC) were purchased from the American Type Culture Collection (ATCC, U.S.A.). Cells were cultured in Endothelial Cell Medium (ECM) (ScienCell, U.S.A.) supplemented with 10% fetal bovine serum (FBS), 2 mM l-glutamine, 5 U/ml penicillin G, and 5 µg/ml streptomycin sulfate (Invitrogen, U.S.A.). In addition, hypoxic HUVECs or HCAECs were treated with 200 µmol/ml CoCl_2_. All cells were cultured at 37°C in an incubator with 5% CO_2_ and 95% humidity. The hsa-miR-24-3p mimic (miR-24-3p) (5′-UGGCUCAGUUCAGCAGGAACAG-3′), the hsa-miR-24-3p inhibitor mimic (miR-24-3p inhibitor) (5′-CUGUUCCUGCUGAACUGAGCCA-3′), and the corresponding negative control (miR-NC) (5′-UUCUCCGAACGUGUCACGUTT-3′) were synthesized by GenePharma (Suzhou, Jiangsu, China).

### Cell transfection

Hypoxic HUVECs at a density of 0.5 × 10^6^/well were inoculated into a six-well plate (Beyotime, China) and incubated at 37°C and 5% CO_2_ overnight when the cells reached 60–80% confluence. According to the protocol, the cell medium was replaced with a serum-free culture medium, followed by transfection with the Lipofectamine™ 3000 Transfection Reagent (Invitrogen, U.S.A.). After 6 h of transfection, the serum-free medium was replaced with ECM containing 10% FBS.

### Quantitative real-time polymerase chain reaction

Total RNA was isolated from cells using TRIzol reagent (Invitrogen, U.S.A.) as previously described [24,25]. Then, reverse transcription was performed by using cDNA Synthesis Kits (Invitrogen, U.S.A.). For quantitative real-time polymerase chain reaction (RT-qPCR), we used the following primer sets for hypoxia-inducible factor-1α (HIF1A): HIF1A-F, 5′‐TCAAGTCAGCAACGTGGAAG‐3′ and HIF1A-R, 5′‐TATCGAGGCTGTGTCGACTG‐3′. The primer sets for NKAP: NKAP-F, 5′‐GTATCCCACGAAGAGGTGAAAT‐3′ and NKAP-R, 5′‐ATGCCTGCTACCACTCATTAC‐3′. RT-qPCR was performed using SYBR™ Green PCR Master Mix (Applied Biosystems, U.S.A.). miR-24-3p and mRNA expression levels were normalized to U6 and GAPDH, respectively, and the gene expression fold changes were calculated by relative quantification (2^−ΔΔ*C*_t_^).

### Cell counting kit-8 assay

The cell counting kit-8 (CCK-8) assay was performed as previously described [23]. Hypoxic HUVECs were transfected with si-NC, si-NKAP+miR-NC or si-NKAP+miR-24-3p. The cells at a density of 3 × 10^4^ cells/ml, 100 μl/well, were added to 96-well plates and incubated in a 5% CO_2_ incubator at 37°C for Days 1, 2, 3, and 4. Live cells were measured with the Cell Counting Kit-8 reagent (Dojindo, Japan) following the manufacturer’s instructions. After CCK-8 reagent was added, readings were performed at 450 nm.

### Migration and invasion assays

The cells were transfected with miR-NC or miR-24-3p. For the transwell migration assay, 1 × 10^5^ cells were plated on to the top chamber containing a noncoated membrane. For the Transwell invasion assay, 3 × 10^5^ cells were seeded into the top chamber precoated with Matrigel. Transwell assays were performed using a 24-well cell Transwell assay kit (Cell Application, U.S.A.) according to the manufacturer’s manual. Briefly, after 24 h, the membranes were fixed with 95% alcohol for 20 min and then stained with Hematoxylin for another 10 min. Six random fields were counted for each insert, and the data are presented for three independent experiments.

### Luciferase reporter assay

A luciferase reporter assay was performed as described previously [25]. Briefly, the luciferase reporter vector pGL3-basic (Addgene) was used to generate a luciferase reporter construct. Wildtype and mutant NKAP fragments containing the KpnI and XhoI restriction enzyme cutting sequences were synthesized. The synthesized DNA was digested with KpnI and XhoI, followed by insertion into the pGL3-basic vector. Next, hypoxic HUVECs were seeded at a density of 1×10^6^ cells/well in six-well plates. Then, hypoxic HUVECs were cotransfected with pGL3-NKAP-WT and miR-24-3p or pGL3-NKAP-MUT and miR-24-3p. The cells were maintained in an incubator for 24 h. Finally, relative luciferase activity was determined by a dual luciferase assay (Promega, U.S.A.).

### Western blotting analysis

Hypoxic HUVECs were transfected with miR-NC and miR-24-3p. After 48 h, total proteins were extracted, and quantitation by the BCA protein assay was performed. Then, 30 μg/well protein was separated by 10% SDS/PAGE and transferred to PVDF membranes. First, the membranes were blocked with 5% nonfat milk, and then anti-HIF1A, anti-NKAP, anti-NF-κB, and anti-pro-IL-1β antibodies were added as primary antibodies. Next, the membranes were incubated at 4°C overnight and washed three times. Next, the membranes were incubated with secondary antibodies at room temperature for 1 h and washed another three times, after which chemiluminescence detection was performed according to the manufacturer’s instructions. β-tubulin was used as an endogenous protein for normalization.

### Statistical analysis

All data, collected from three independent experiments, are expressed as the mean ± SD and were processed using GraphPad Prinsm8 software. Student’s *t* test or one-way ANOVA was used to estimate the differences among the groups. *P*<0.05 was considered to be statistically significant.

## Results

### In AMI patients, the expression level of miR-24-3p is down-regulated, and the expression level of IL-1β or NKAP is up-regulated, which are negatively correlated

**Figure 1:**
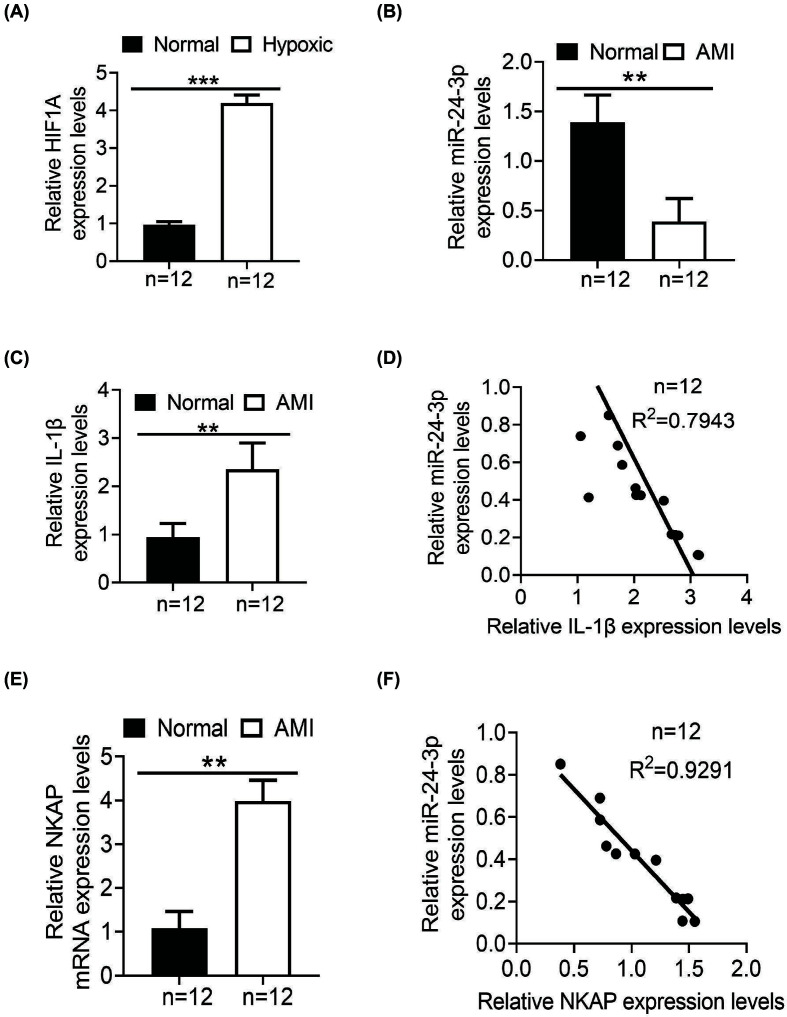
The expression levels of miR-24-3p and IL-1β in AMI patients (**A**) qRT-PCR was used to verify the expression of HIF1A in hypoxic HUVECs 48 h after treatment with 200 µmol/ml CoCl_2_. (**B**) qRT-PCR was used to analyze the expression levels of miR-24-3p in 12 paired samples (*n*=12). (**C**) The expression levels of IL-1β in 12 paired samples were detected by ELISA (*n*=12). (**D**) A negative correlation existed between the miR-24-3p level and the IL-1β protein level (*n*=12). (**E**) The expression levels of NKAP in 12 paired samples (CECs) were detected by qRT-PCR (*n*=12). (**F**) A negative correlation existed between the miR-24-3p level and the NKAP mRNA level (*n*=12). All the experiments were repeated at least three times. Data represent the mean ± SD; **P<0.01, ***P<0.001 compared with the control.

HIF1A is induced and activated by hypoxia, a direct reflection and an important sign of tissue hypoxia [26]. Additionally, our previous data showed that miR-24 inhibits the proliferation of HUVECs [22], which led us to hypothesize that miR-24-3p could affect hypoxic HUVECs and play an important role in angiogenesis. To examine the possible effects of miR-24-3p on hypoxic HUVECs *in vitro*, we used CoCl_2_-inducible hypoxic HUVECs as a model in the present study. HUVECs were pretreated with 200 μmol/ml CoCl_2_ for 24 h. After transfection with miR-NC or miR-24-3p, the effect of CoCl_2_ on HIF1A mRNA in HUVECs was analyzed by RT-qPCR, with GAPDH used as an internal control. The levels of HIF1A mRNA in miR-24-3p were increased by 4.2-fold compared with miR-NC (Figure 1A).

Furthermore, we analyzed the expression levels of miR-24-3p and IL-1β in blood samples from 12 healthy donors and 12 AMI patients using real-time PCR and ELISA, respectively. It has been reported that miR-24-3p is down-regulated in a myocardial I/R injury mouse model [3]. Interestingly, the relative levels of miR-24-3p expression were significantly lower in the AMI samples than in the normal samples (Figure 1B). In contrast, significantly higher levels of IL-1β expression in the AMI samples were found relative to the levels in the normal samples (Figure 1C). In addition, we observed a negative correlation between the miR-24-3p level and IL-1β protein level (Figure 1D). Interestingly, significantly higher levels of NKAP in the AMI samples (CECs) were found relative to the levels in the normal samples (Figure 1E). In addition, we observed a negative correlation between the miR-24-3p level and NKAP mRMA level (Figure 1F).

### IL-1β promotes hypoxic HUVECs proliferation

**Figure 2:**
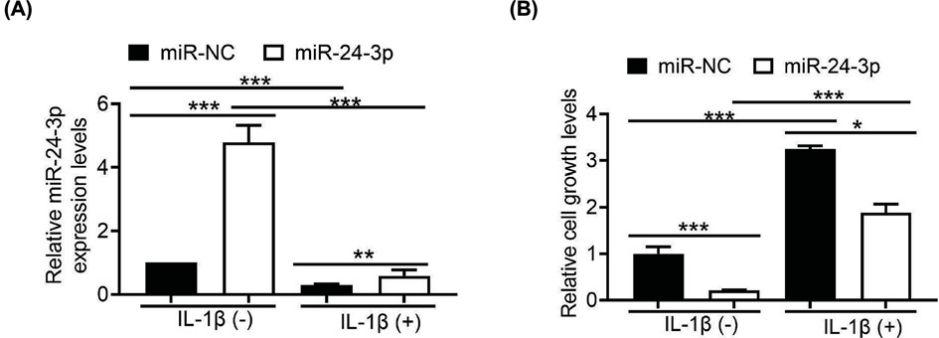
IL-1β promotes hypoxic HUVECs proliferation (**A**) qRT-PCR was used to verify the expression of miR-24-3p in hypoxic HUVECs 48 h after transfection with 10 nM miR-24-3p or miR-NC (with or without IL-1β). (**B**) The proliferation of cells transfected with miR-24-3p or miR-NC (with or without IL-1β). All the experiments were repeated at least three times. Data represent the mean ± SD; *P<0.05, **P<0.01, ***P<0.001 compared with the control.

To investigate the function of miR-24-3p in hypoxic HUVECs, miR-24-3p was overexpressed in hypoxic HUVECs by transient transfection with miR-24-3p and miR-NC mimics. Transfection efficiency was monitored by RT-qPCR. The relative expression levels of miR-24-3p were significantly higher in the miR-24-3p-transfected HUVECs than in the control HUVECs (Figure 2A). It has previously been reported that miR-24-3p plays an essential role in inhibiting HUVECs proliferation [27]. We therefore performed a CCK-8 assay to explore the effect of miR-24-3p on hypoxic HUVECs proliferation. As expected, hypoxic HUVECs transfected with miR-24-3p had a lower level of proliferation than cells transfected with miR-NC (Figure 2B). Recently, emerging evidence has confirmed that IL-1β is up-regulated in AMI patients [28]. Therefore, to explore whether miR-24-3p activity was present in hypoxic HUVECs treated with IL-1β, we used RT-qPCR to determine the level of miR-24-3p when transfected with miR-NC or miR-24-3p. IL-1β reduced miR-24-3p mRNA levels in both the miR-NC and miR-24-3p groups compared with the control group without 1β (Figure 2A). In addition, the results showed that IL-1β increased hypoxic HUVECs growth in both the miR-NC and miR-24-3p groups compared with the control group without 1β (Figure 2B). Interestingly, in hypoxic HUVECs treated with IL-1β, the growth rate was significantly up-regulated by miR-NC transfection compared with miR-24-3p transfection. Therefore, we concluded that IL-1β promotes hypoxic HUVECs proliferation (Figure 2), likely due to the decrease in miR-24-3p.

### IL-1β promotes migration and invasion in hypoxic HUVECs

**Figure 3:**
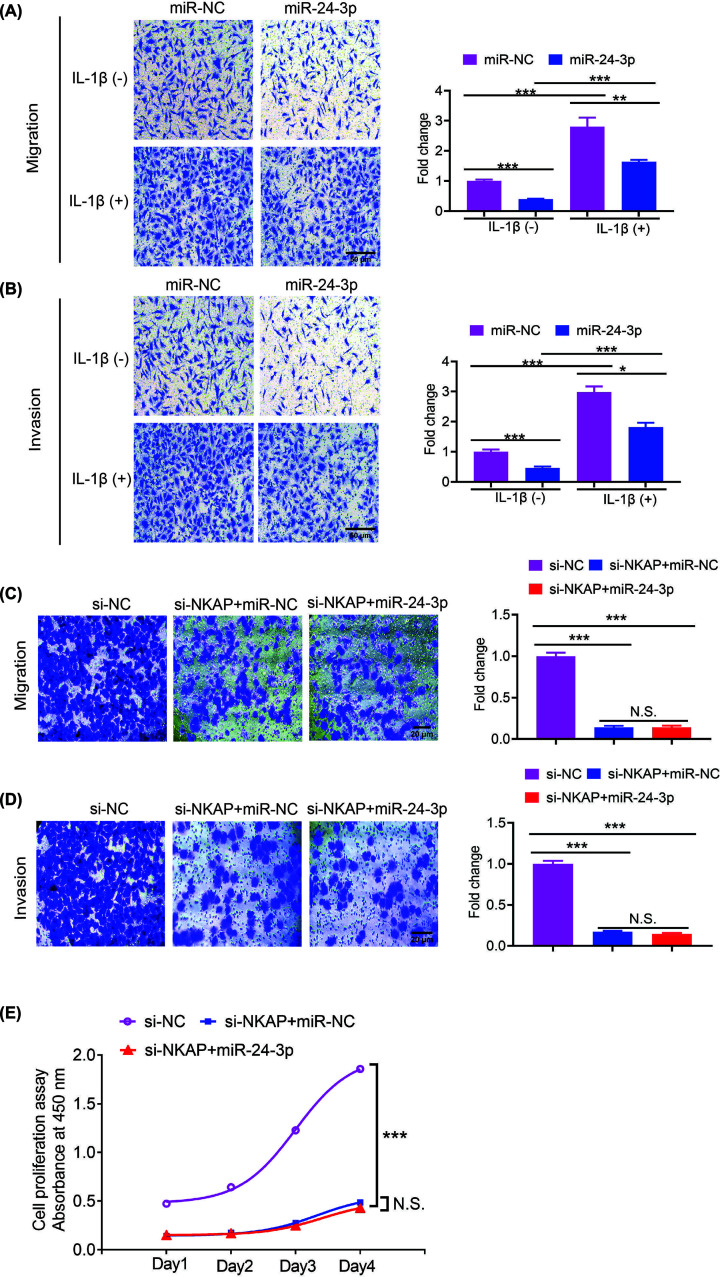
IL-1β significantly promotes hypoxic HUVECs migration and invasion (**A**) Hypoxic HUVECs transfected with miR-24-3p or miR-NC were subjected to migration assays (with or without IL-1β). The migrated cells were counted, and the relative fold change is displayed in a histogram on the right. (**B**) Hypoxic HUVECs transfected with miR-24-3p or miR-NC were subjected to an invasion assay (with or without IL-1β). The number of invading cells was counted, and the relative fold change is shown in a histogram on the right. (**C**) Hypoxic HUVECs transfected with si-NC, si-NKAP+miR-NC or si-NKAP+miR-24-3p were subjected to migration assays. The migrated cells were counted, and the relative fold change is displayed in a histogram on the right. (**D**) Hypoxic HUVECs transfected with si-NC, si-NKAP+miR-NC or si-NKAP+miR-24-3p were subjected to an invasion assay. The number of invading cells was counted, and the relative fold change is shown in a histogram on the right. (**E**) Hypoxic HUVECs transfected with si-NC, si-NKAP+miR-NC or si-NKAP+miR-24-3p were subjected to a CCK-8 assay. All of the experiments were repeated at least three times. Data represent the mean ± SD; *P<0.05, **P<0.01, ***P<0.001 compared with the control.

To investigate the effects of IL-1β on cell migration and invasion, we conducted cell migration and invasion assays. Hypoxic HUVECs were transfected with miR-24-3p and miR-NC mimics. The migration and invasion abilities of hypoxic HUVECs were determined by Transwell assays. The results showed that cells treated with miR-24-3p showed lower levels of migration and invasion than those treated with miR-NC without IL-1β (Figure 3A,B). Similarly, cells treated with miR-24-3p showed lower levels of migration and invasion than those treated with miR-NC and IL-1β (Figure 3A,B). However, higher levels of migration and invasion were observed in cells treated with IL-1β than in those without IL-1β (Figure 3A,B). These data indicated that IL-1β significantly promotes the migration and invasion of hypoxic HUVECs and that miR-24-3p partially rescues hypoxic HUVECs migration and invasion.

Furthermore, to investigate whether miR-24-3p functions through NKAP, we conducted cell migration and invasion assays, hypoxic HUVECs were transfected with si-NC, si-NKAP+miR-NC, or si-NKAP+miR-24-3p. The migration and invasion of hypoxic HUVECs were determined by Transwell assays. The results showed that the cells treated with si-NKAP+miR-NC or si-NKAP+miR-24-3p showed lower levels of migration and invasion than those treated with si-NC (Figure 3C,D). However, no difference in migration and invasion was observed in the cells treated with si-NKAP+miR-NC or si-NKAP+miR-24-3p. Similarly, the cells treated with si-NKAP+miR-NC or si-NKAP+miR-24-3p showed lower levels of proliferation than those treated with si-NC (Figure 3E), and no difference in proliferation was observed in the cells treated with si-NKAP+miR-NC or si-NKAP+miR-24-3p. These data indicated that knockdown NKAP significantly suppresses the migration and invasion, proliferation of hypoxic HUVECs and that miR-24-3p suppresses hypoxic the migration and invasion, proliferation of HUVECs through NKAP.

### miR-24-3p directly targets the 3′-UTR of NKAP in hypoxic HUVECs

**Figure 4:**
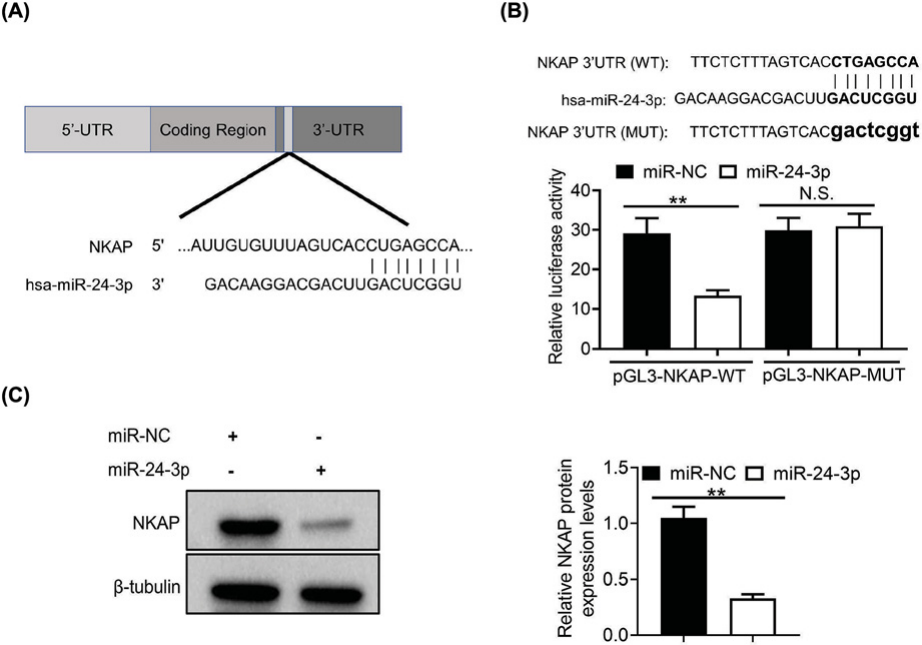
miR-24-3p directly targets NKAP in hypoxic HUVECs (**A**) The predicted binding of miR-24-3p with the NKAP 3′-UTR. (**B**) A dual-luciferase reporter assay was performed to confirm NKAP as the miR-24-3p target in hypoxic HUVECs. NKAP linked to the firefly luciferase reporter were transfected into hypoxic HUVECs. The *Renilla* luciferase reporter construct pRL-TK was also used in the co-transfection to monitor the transfection efficiency. Relative luciferase activity was firefly luciferase expression was normalized to *Renilla* luciferase. (**C**) Western blotting was performed to validate the NKAP protein levels in hypoxic HUVECs transfected with miR-24-3p or miR-NC. The protein expression level, quantified by band intensity and normalized to β-tubulin, is displayed in the right panel. All of the experiments were repeated at least three times. Data represent the mean ± SD; N.S., not significant, **P<0.01.

To determine the mechanism by which miR-24-3p affects hypoxic HUVECs, the binding site of miR-24-3p was estimated to be within the 3′-UTR of NKAP by the TargetScan Human 7.2 database [29]. Thus, we identified NKAP as a potential target gene of miR-24-3p. Furthermore, the target sequences showed that the miR-24-3p target sites were highly conserved (Figure 4A). To validate whether NKAP was the target of miR-24-3p, we generated the wildtype and mutated 3′ UTRs of the NKAP gene that contained the binding sequences for miR-24-3p. Briefly, WT NKAP or MUT NKAP reporters were co-transfected into hypoxic HUVECs with miR-24-3p or miR-NC, respectively. The results showed that miR-24-3p significantly reduced the relative luciferase activity in NKAP-WT-transfected hypoxic HUVECs compared with miR-NC (Figure 4B). In addition, when miR-24-3p and miR-NC were co-transfected with the MUT reporter of NKAP, there was no significant difference in relative luciferase activity between the two groups (Figure 4B). To explore whether miR-24-3p affected the protein expression levels of NKAP in hypoxic HUVECs, the expression levels of NKAP were evaluated in hypoxic HUVECs following transfection with miR-24-3p or miR-NC. The data showed that miR-24-3p dramatically decreased NKAP protein expression levels (Figure 4C). These results indicated that miR-24-3p regulates NKAP gene expression by directly targeting the 3′-UTR of NKAP mRNA.

### Inhibition of miR-24-3p restores NKAP/NF-κB repression by IL-1β

**Figure 5:**
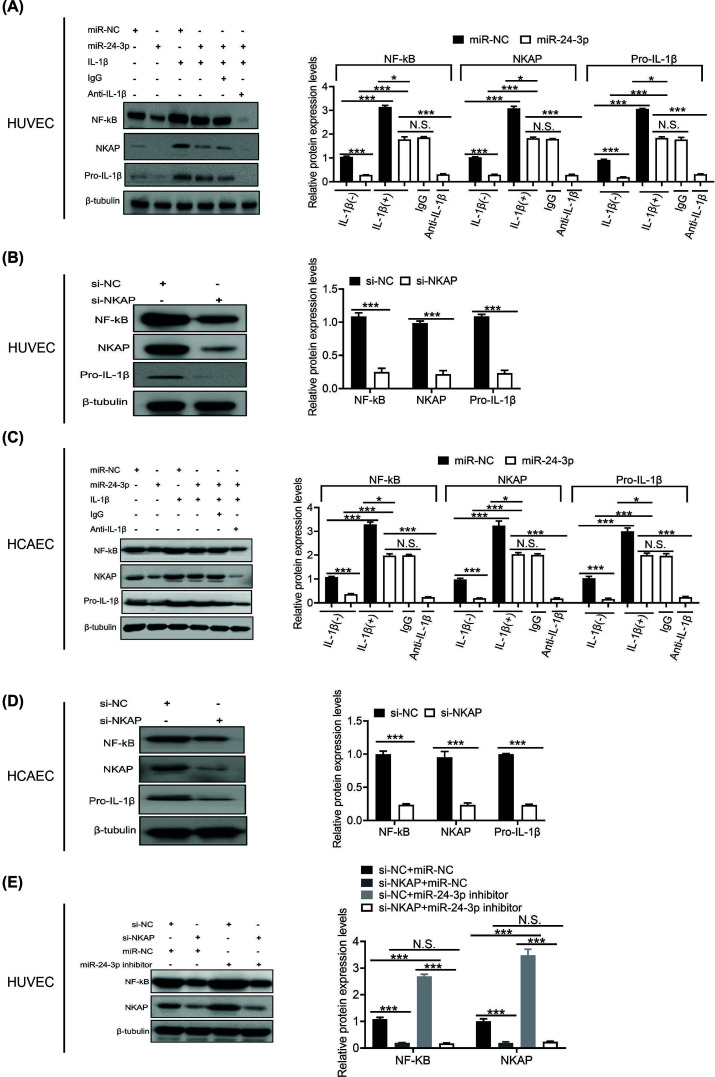
Inhibition of miR-24-3p restores NKAP/NF-κB/Pro-IL-1β by IL-1β (**A**) Western blot analysis of NKAP/NF-κB/Pro-IL-1β protein expression levels in hypoxic HUVECs transfected with miR-24-3p or miR-NC (with or without IL-1β). (**B**) Western blot analysis of NKAP/NF-κB/Pro-IL-1β protein expression levels in hypoxic HUVECs transfected with miR-NC or miR-24-3p (with or without IL-1β). (**C**) Western blot analysis of NKAP/NF-κB/Pro-IL-1β protein expression levels in hypoxic HCAECs transfected with miR-24-3p or miR-NC (with or without IL-1β). (**D**) Western blot analysis of NKAP/NF-κB/Pro-IL-1β protein expression levels in hypoxic HCAECs transfected with miR-NC or miR-24-3p (with or without IL-1β). (**E**) Western blot analysis of NKAP/NF-κB protein expression levels in hypoxic HCAECs cotransfected with si-NC+miR-NC, si-NKAP+miR-NC, si-NC+miR-24-3p inhibitor or si-NKAP+miR-24-3p inhibitor. All the experiments were repeated at least three times. Data represent the mean ± SD; N.S., not significant, *P<0.05, ***P<0.001 compared with the control.

NKAP is involved in IL-1-induced NF-κB activation [30]. In addition, NKAP is up-regulated in many kinds of tumors [31], such as colon cancer [32], hepatocellular carcinoma [33], breast cancer [34], and so on. On the other hand, NKAP knockdown also reduces the proliferation of cancers [35]. Therefore, to understand how IL-1β regulates miR-24-3p/NKAP/NF-κB/Pro-IL-1β activities in hypoxic HUVECs, we conducted a western blot analysis of these three proteins in hypoxic HUVECs following transfection with miR-24-3p and miR-NC. The results showed that IL-1β dramatically increased NKAP/NF-κB/Pro-IL-1β protein expression levels after transfection with miR-NC compared with transfection with miR-24-3p. As we expected, the effect of IL-1β on the NKAP/NF-κB/Pro-IL-1β protein expression level was specific, as it could be blocked by anti-IL-1β antibody but not by the control antibody (Figure 5A). Moreover, depletion of NKAP in hypoxic HUVECs also significantly decreased the expression of NF-κB/Pro-IL-1β (Figure 5B). Consistent with the hypoxic HUVEC system, the hypoxic HCAEC system showed similar results (Figure 5C,D). Furthermore, to determine whether miR-24-3p can regulate other protein-coding genes that may regulate NF-κB, we conducted a western blot analysis of NF-κB proteins in hypoxic HUVECs following cotransfection with si-NC+miR-NC, si-NKAP+miR-NC, si-NC+miR-24-3p or si-NKAP+miR-24-3p. The results showed that the effect of miR-24-3p on the NF-κB protein expression level was dependent on NKAP, as it could be blocked by si-NKAP but not by the control si-NC (Figure 5E). These data also demonstrated that the NF-κB/Pro-IL-1β pathway is a downstream functional regulator of miR-24-3p. Moreover, IL-1β inhibition of miR-24-3p restores NKAP/NF-κB/Pro-IL-1β repression.

## Discussion

Angiogenesis is a physiological process [36]. New blood vessels form from pre-existing vessels, which are formed in the earlier phases of vasculogenesis [37]. It has been demonstrated that angiogenesis plays a critical role in regulating different kinds of cellular processes [38], including proliferation, metastatic spread, migration, and invasion [39]. Recently, it has been demonstrated that miR-24-3p dysregulation in prostate cells promotes microvascular proliferation of endothelial cells (ECs) [40]. Moreover, inhibition of miR-24-3p in the microvasculature restores angiogenesis [17].

Furthermore, it has been reported that recombinant IL-1α significantly enhances HUVEC growth and tube-like formation [41]. IL-1β is in the same family as IL-1α [42]. Moreover, the biological basis for IL-1β has not yet been clarified. In addition, whether IL-1β-mediated miR-24-3p is involved in hypoxic HUVECs is not known. The present study demonstrated that miR-24-3p expression was significantly lower in the AMI samples than in the normal samples (Figure 1B). Furthermore, IL-1β in the AMI samples was considerably higher than that in the normal samples (Figure 1C). In addition, we observed a negative correlation between the miR-24-3p and IL-1β expression levels (Figure 1D). Additionally, we discovered that IL-1β promoted hypoxic HUVECs proliferation (Figure 2), likely due to the suppression of miR-24-3p. Interestingly, IL-1β significantly promoted the migration and invasion of hypoxic HUVECs; miR-24-3p partially rescued the migration and invasion of hypoxic HUVECs (Figure 3). These data suggest that IL-1β-mediated suppression of miR-24-3p activity may occur in hypoxic HUVECs. Furthermore, the molecular mechanisms underlying the IL-1β effect on miR-24-3p activity appear to be different, further confirming the importance of the cellular environment in the activity of miR-24-3p.

miRNAs are a type of small noncoding RNA that targets protein-coding mRNAs, leading to suppression of gene expression at the post-transcriptional level [43]. miR-24-3p has been reported to be involved in different biological pathways that can affect HUVEC proliferation, migration, and invasion [17]. Therefore, it is critical to understand the involvement of miR-24-3p in hypoxic HUVECs. However, the biological basis for the supposed prognostic impact of miR-24-3p in hypoxic HUVECs has not yet been clarified. In the present study, by bioinformatics analysis (TargetScan 7.2), NKAP was found to be the target gene of miR-24-3p, as confirmed by luciferase assay and western blotting. In addition, NKAP is well known for playing a crucial role in antiangiogenic functions [44]. NKAP has also been found to be important in NF-κB activity [30]. NKAP is involved in regulating the growth and invasion of colon cancer cells [32]. However, the function of NKAP in hypoxic HUVECs is unclear. Here, we showed that miR-24-3p dramatically decreased NKAP protein expression levels. These results indicated that miR-24-3p regulates NKAP gene expression by directly targeting the 3′-UTR of NKAP mRNA. Similarly, knockdown of NKAP in hypoxic HUVECs suppressed the NF-κB/Pro-IL-1β signaling pathway. These results indicate that miR-24-3p inhibits hypoxic HUVEC proliferation and migration/invasion by inhibiting NKAP.

NKAP was initially reported as a possible regulator of NF-κB activation, and NKAP is a nuclear protein that activates NF-κB [45]. In this study, we found that IL-1β treatment induced the expression of NKAP in hypoxic HUVECs. Furthermore, we revealed that miR-24-3p regulated the NF-κB pathway by targeting NKAP. The present results showed that miR-24-3p has beneficial effects on the IL-1β-induced proinflammatory response. Taken together, IL-1β promotes hypoxic HUVEC growth through the miR-24-3p/NKAP/NF-κB axis.

## Perspectives

- In AMI patients, miR-24-3p expression is down-regulated, IL-1β or NKAP expression is up-regulated, and the expression of IL-1β or NKAP molecules is negatively correlated with miR-24-3 expression.
- IL-1β promotes hypoxic HUVECs proliferation/migration and invasion by down-regulating miR-24-3p.
- Overexpression of miR-24-3p can partially rescue hypoxic HUVECs proliferation/migration and invasion.
- NKAP is a novel target of miR-24-3p.
- Inhibition of miR-24-3p restores NKAP/NF-κB repression by IL-1β.

## Data Availability

All the data are available on contacting the corresponding authors.

## Abbreviations

AMI: acute myocardial infarction
CCK-8: cell counting kit-8
CEC: circulating endothelial cell
EC: endothelial cell
ECM: Endothelial Cell Medium
FBS: fetal bovine serum
HCAEC: human coronary artery endothelial cell line
HF: heart failure
HIF1A: hypoxia-inducible factor-1α
HUVEC: human umbilical vein endothelial cell
IL-1β: interleukin 1β
miRNA: microRNA
NKAP: NF-κB-activating protein
PBMC: peripheral blood mononuclear cell
RT-qPCR: quantitative real-time polymerase chain reaction
3′-UTR: 3′-untranslated region
